# DeadEasy Mito-Glia: Automatic Counting of Mitotic Cells and Glial Cells in *Drosophila*

**DOI:** 10.1371/journal.pone.0010557

**Published:** 2010-05-10

**Authors:** Manuel Guillermo Forero, Anabel R. Learte, Stephanie Cartwright, Alicia Hidalgo

**Affiliations:** Neurodevelopment Group, School of Biosciences, University of Birmingham, Birmingham, United Kingdom; Columbia University, United States of America

## Abstract

Cell number changes during normal development, and in disease (e.g., neurodegeneration, cancer). Many genes affect cell number, thus functional genetic analysis frequently requires analysis of cell number alterations upon loss of function mutations or in gain of function experiments. *Drosophila* is a most powerful model organism to investigate the function of genes involved in development or disease *in vivo*. Image processing and pattern recognition techniques can be used to extract information from microscopy images to quantify automatically distinct cellular features, but these methods are still not very extended in this model organism. Thus cellular quantification is often carried out manually, which is laborious, tedious, error prone or humanly unfeasible. Here, we present DeadEasy Mito-Glia, an image processing method to count automatically the number of mitotic cells labelled with anti-phospho-histone H3 and of glial cells labelled with anti-Repo in *Drosophila* embryos. This programme belongs to the DeadEasy suite of which we have previously developed versions to count apoptotic cells and neuronal nuclei. Having separate programmes is paramount for accuracy. DeadEasy Mito-Glia is very easy to use, fast, objective and very accurate when counting dividing cells and glial cells labelled with a nuclear marker. Although this method has been validated for *Drosophila* embryos, we provide an interactive window for biologists to easily extend its application to other nuclear markers and other sample types. DeadEasy MitoGlia is freely available as an ImageJ plug-in, it increases the repertoire of tools for *in vivo* genetic analysis, and it will be of interest to a broad community of developmental, cancer and neuro-biologists.

## Introduction

*Drosophila* is a most powerful model organism to analyse gene function in vivo. Fluorescence labelling and laser scanning confocal microscopy are employed routinely by many *Drosophila* labs to visualise and analyse cellular features in embryos, larvae and adult fruit-flies, because they enable capturing 3D images by focusing through increasing depths. Generally phenotypic analyses are carried out by qualitative and manual inspection of images. There is great potential in extracting information from the large collections of images resulting from confocal microscopy, and image processing and pattern recognition methods can be used to automatically quantify information (e.g. number of cells) from such images. Surprisingly, not a lot has been done to date to enable automatic measurements from Drosophila using image processing solutions. We have been addressing the question of how to obtain accurate, simple and fast quantitative information on cell number in vivo [1], [2]. Knowing the number of dividing cells or glial cells in a Drosophila embryo can be important, e.g. to address questions relating to the control of growth and nervous system development, the functions of growth factors in vivo, and the consequences of abnormal gene function that may result in tumorous growth, including gliomas.

The identification and counting of cells is a difficult task both for human, manual counters and for image processing. Samples are analysed by observing each image of a stack and verifying whether an object positive for the marker used is a cell or not. There are several sources of error, related to the properties of the lenses, the detector and the fluorescence [3], large variations in image contrast, thickness of the sample and irregular staining of objects. Projections of sections cannot be used since they obscure individual cells. However, in 3D a cell may change considerably in intensity, shape or distribution across images in the stack, making reference points difficult to establish. Similar to astronomical images, the objects of interest (i.e. cells) are fuzzy and they do not have clearly defined borders, so in many cases cells are difficult to distinguish, introducing a subjective assessment by the observer of whether an object is a cell or not. As a result, objects having similar intensities and sizes are counted as cells in some images, but rejected in others. Thus error can be due to variations in cell size and morphology, and in signal intensity.

The problem of counting cells from confocal microscopy images has been addressed before [4], [5]. For the analysis of 2D images, a method for segmenting grayscale images of corneal endothelial tissue used a dome extractor based on morphological grayscale reconstruction and marker-driven watershed segmentation, yielding binary images of the corneal cell network [6], [7]. A technique was developed for counting Drosophila brain nuclei, but several parameters must still be adjusted manually [8]. There is a technique that combines morphological filtering, the gradient of the images and the data's shape to segment cells in 2D and 3D images, but it is time consuming and requires good image contrast [9], [10], [11]. Automatic techniques have been developed to segment cell nuclei from tissue sections or whole Drosophila brains in 2D and 3D images, but they require intensive computation, making them unsuitable for large sample sizes. Cell Profiler software [12] allows users to combine image-processing methods to develop techniques to count cells, but this requires some knowledge of computation and it has not been tested for specific applications. Several methods have been developed in order to identify dividing cells in microscopy: a 2D method to track dividing cells in living fungal networks [13]; and a method for the determination of mitotic delays in human cells [14], [15], which however is applied to 2D Maximum Intensity Projections of the stack. However, these methods do not answer the question of how many mitotic cells there are per stack.

Counting cells in Drosophila is a complex task, due the variability in image qualities given by the different properties of each cell marker [1], [2]. We present here a new method that employs image filtering and mathematical morphology techniques to count dividing cells labelled with anti-phospho-Histone-H3 and glial cells labelled with the nuclear marker anti-Repo, from images acquired by confocal microscopy. This programme belongs to the DeadEasy suit of methods that we are developing to count the number of different cell types in Drosophila: we have previously shown that DeadEasy Caspase counts accurately the number of dying cells in vivo [2], and DeadEasy Neurons counts automatically the number of neuronal nuclei in vivo [1]. Having separate methods for each cell type and marker is paramount for accuracy. The difference between the DeadEasy programmes lies in the methods developed to segment and distinguish the objects of interest, which depend on the characteristic of the cell marker employed to label the cells. Together, these methods expand the repertoire of technical approaches for functional genetic in vivo analysis using Drosophila.

## Materials and Methods

### Genetics

The stock used as wild-type was *y w*. Mutants: i) wt = y w; i) *pros^J013^/TM6B lacZ*. iii) *cycE^AR95^*/CyO lacZ. Mutant embryos were identified by the absence of anti-βgal signal when staining the embryonic population with anti- β gal antibodies.

### Immunohistrochemistry and laser scanning confocal microscopy

**Figure 1:**
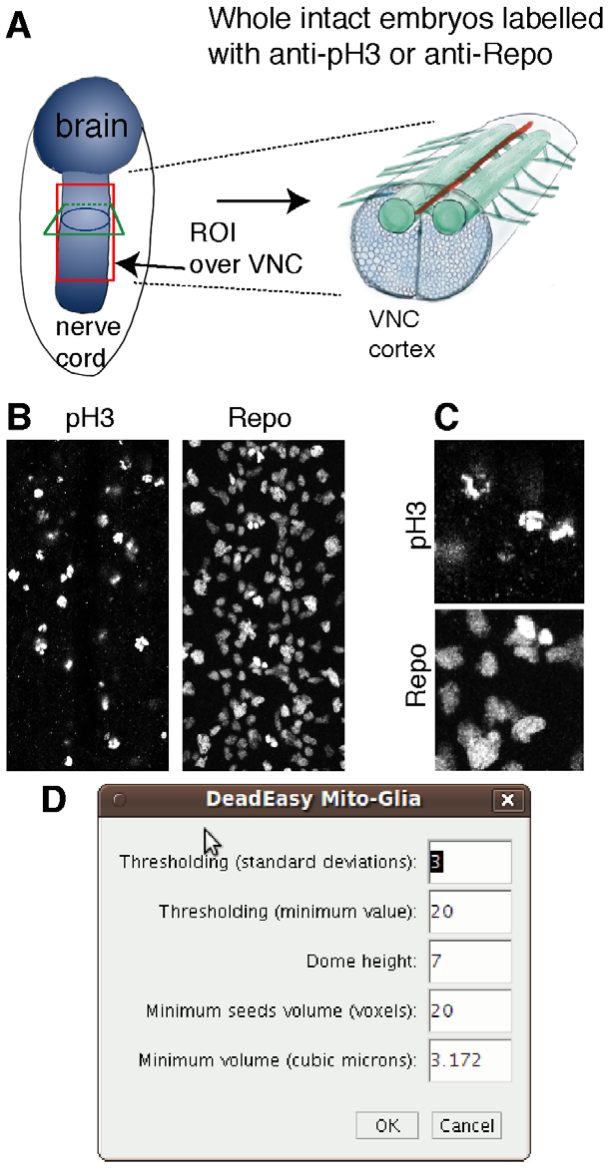
Cell division and glia in the embryonic VNC. (A) Diagram showing an embryo (left) and a cross-section view of the ventral nerve cord (VNC, right). The red box indicates an example of a region of interest (ROI) comprising the VNC and excluding the epidermis; any ROI of choice can be used. (B) Characteristic embryonic VNCs labelled with the mitotic marker pH3 and the glial marker Repo. (C) Higher magnification views of details from specimens in (B) to show the properties of the images. (D) Interactive window to enable users to change the parameters to apply the programme to other markers or sample types.

*Drosophila* embryos were stained with rabbit anti-pHistone-H3 antibodies to visualise mitotic cells (at 1∶300 Upstate Biotechnology) or mouse anti-Repo antibodies (at 1∶100 Developmental Studies Hybridoma Bank, Iowa), to visualise glial nuclei antibodies using standard methods (Figure 1A). Antibody labelled cells were detected with anti-Rabbit or anti-Mouse secondary antibodies, directly conjugated to the fluorochrome Alexa-488. Specimens were mounted in Vectashield (Vector Labs).

Mounted whole embryos were scanned using a BioRad Radiance 2000 or Leica TCS-SP2-AOBS laser scanning confocal microscopes. The settings at the confocal microscope need to be fixed for all samples and acquisition has to be set ensuring that the dynamic range of the histogram covers all grey values. The conditions for scanning were 60× lens, no zoom and 0.5µm slice step, acquisition resolution of 512×512 pixels. Fixed iris (pinhole), laser intensity, gain and offset were maintained throughout all samples of the same experiment. Software algorithm was developed and evaluated using Java and ImageJ under Ubuntu Linux platform in a PC Pentium 4 running at 3 GHz with 1.5 GB RAM.

### Mathematical Algorithm

DeadEasy MitoGlia was developed to count cells stained with either anti-phospho-Histone-H3 (pH3) or anti-Repo (Repo) antibodies in embryos (Figures 1A–C). The image characteristics are (Figure 1B,C): (1) sparsely distributed embryonic nuclei; (2) nuclei can appear connected and must be separated; (3) as pH3 stains chromosomes, shape can be irregular, although it is rather regular when using Repo; (4) non specific background is low; (5) signal intensity is high. To determine which parameters characterize pH3 stained cells, a sample of 100 p-H3 cells obtained from one embryo were studied.

3D image processing techniques can be employed to process a stack of images in order to improve the quality of segmentation. This is important where the signal to noise ratio is low, as some particles that may appear to be noise in a 2D image, can be recognized as true particles in 3D [16]. Because of florescence attenuation with increasing depth due to finite transparency of the sample, photo-bleaching, and the thickness of the embryo, signal intensity decays with increasing focus depth. Therefore, frequently 3D techniques apply an intensity correction. One of the simplest methods employs the average or the maxima of the foreground of each image as a parameter of intensity attenuation and then applies an inverse function to compensate for the loss [11], [17], [18]. However, these techniques do not provide good results when the background changes abruptly from one image to another, as is common in Drosophila samples. More complex techniques can also be employed, but they are time-consuming [19], [20], [21], [22], [23], [24] or require a more complicated acquisition system [25]. However, if a convenient segmentation technique is applied to each image based specifically on its properties, an intensity correction method can be avoided. Here, 2D image processing techniques are used for grey-scale image processing, and 3D techniques are employed once the intensity of the images is no longer relevant, i.e. after they are binarised, thus gaining speed in the process.

**Figure 2:**
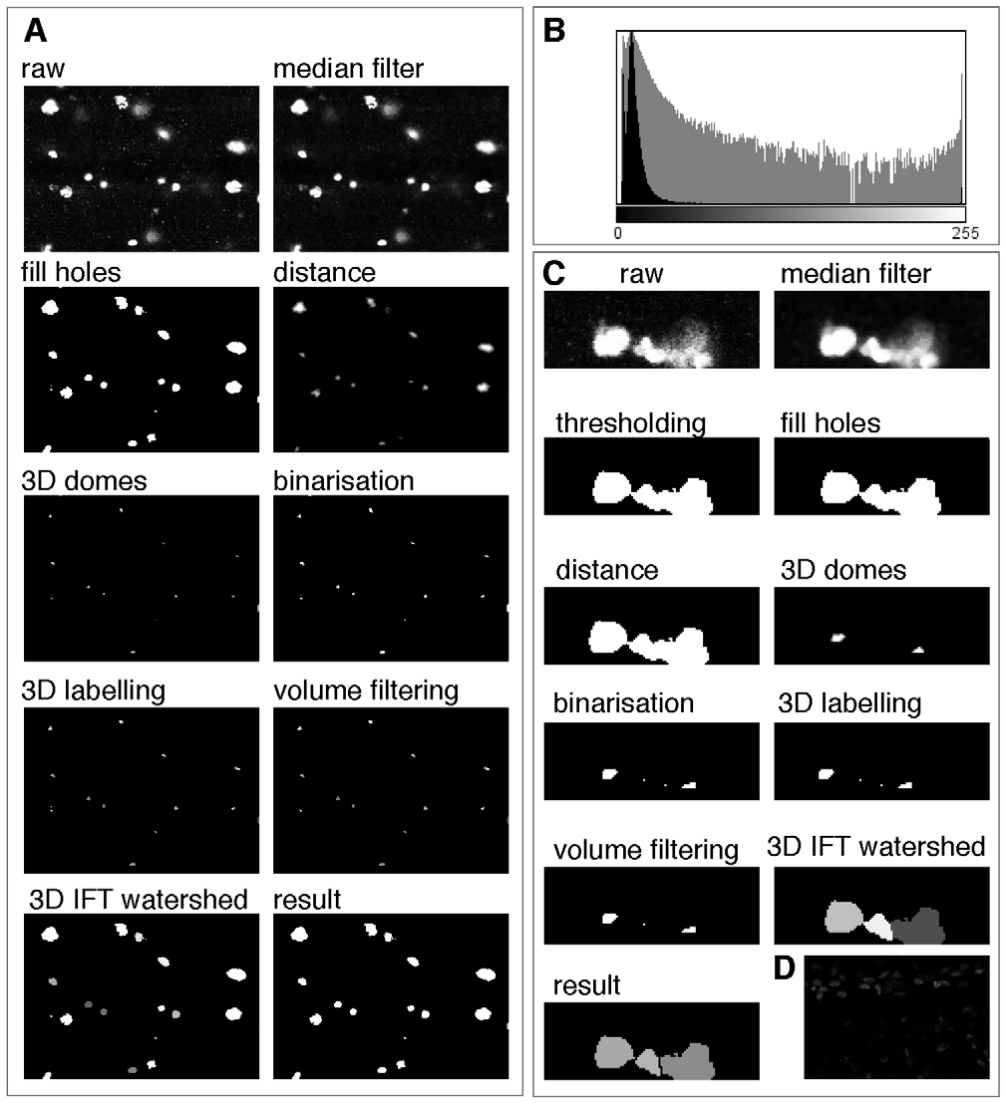
Image processing steps. (A) Images showing the image processing steps starting from the raw image and finishing in the result, which corresponds to the identified objects (cells). (B) Histogram of a typical pH3 stained image. (C) Higher magnification examples to show, as in (A), the different processing steps. This example shows the power of the programme to separate cells that in some slices may appear to be joined. (D) Example of a faintly stained sample that DeadEasy MitoGlia cannot process and must be discarded.

**Figure 3:**
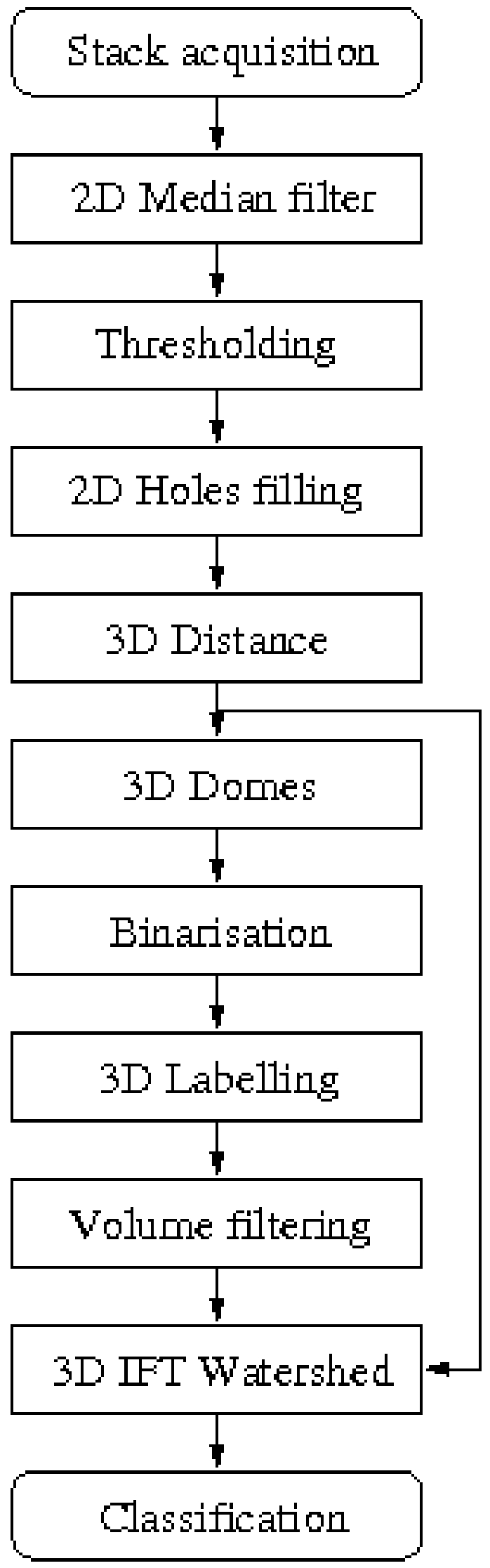
Image processing algorithm. Digrammatic representation of the different image processing steps.

The first segmentation step tries to reduce the noise present in the images. Confocal microscopes employ photon emission to produce images. Given that the number of photons produced is very small, statistical variation in the number of detected photons - which follows a Poison distribution - is the most important source of noise. To reduce noise several non-linear filters can be employed. One of the simplest techniques, the median filter [1], [2], was employed here given that it provided good noise reduction without affecting the borders of the objects on the images (Figure 2A,C and Figure 3).

Given that the cell borders are fuzzy, a thresholding technique is more appropriate than an edge segmentation technique for segmentation. Thresholding techniques allow sorting the pixels of the image as background or foreground. Due to the decrease of the cells' intensity through the stack a threshold value must be found to binarise each image based on the typical histogram of the filtered images (Figure 2A,C and Figure 3). The mode corresponding to the cells is almost imperceptible due to the corresponding small number of pixels compared to the number of background pixels. Given the low number of foreground pixels the histogram can be considered unimodal (Figure 2B). Thresholding techniques are generally not appropriate to binarise unimodal images. Thus, similarly to the method employed by Forero et al. [2] to threshold Caspase images, the background mode was assumed to follow a Gaussian distribution *G(q)* and the pixels belonging to the mitotic cells were considered outliers. The best Gaussian function was found by minimizing the square error between the histogram *h*(*q*) in the interval corresponding to the mode. The threshold value t, to segment the images is given by
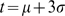
where *μ* and *σ* represent the mean and the standard deviation of the background mode respectively. After thresholding several objects still had some small holes (Figure 2A,C and Figure 3). They were filled with foreground colour by verifying if each hole was surrounded by foreground pixels.

Cells that initially appeared connected were separated by defining the watershed lines between them (Figure 2C and Figure 3). To this end, the first step consisted in marking each cell with a seed. If more than one seed is found per cell, it will be subdivided (i.e. over-segmentation), but if no seed is found the cell will not be recognised. In order to find the seeds a 3D distance transformation was applied. In this way, each voxel of an object takes the value of the minimum distance to the background, and the highest distance will correspond to the furthest point from the borders (Figure 2A,C and Figure 3). Seeds are determined by viewing the stack as a 4D orographic system, where the height of each point is given by the distance of the voxel in that position from the border, and the cells are viewed as peaks or domes of mountains separated by dark valleys [6], [7]. A 3D h-dome operator based on a morphological gray scale reconstruction is applied to extract and mark the cells [7]. The choice of *h* found experimentally was 7, corresponding to the standard minimum distance between the centre of a cell and the voxels surrounding it. Thus, *h* = 7 resulted in marking all cells, which allowed to distinguish cells very close together. However, several seeds could appear in one cell. The h-domes transform of an image *q(x,y)* was obtained by performing a morphological reconstruction of *q(x,y)* from the result of the subtraction *q(x,y)*-*h*, where *h* is a positive scalar, and subtracting the result of the reconstruction from the original image, that is : 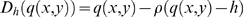 [7] where the reconstruction 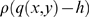 is also known as the h-maxima transform. Subsequently, images were binarised by thresholding the h-domes images [7], resulting in a good identification of the cells (Figure 2A,C and Figure 3). Each seed consists of a set of connected voxels. The h extended-maxima, i.e. the regional maxima of the h-maxima transform, can also be employed to mark the cells [9], [10], [11], but the first procedure produced more domes corresponding to cells. Thus, 3D domes were found. Each seed was labelled employing 18-connectivity.

In order to avoid over-segmentation after watershed, redundant seeds must be eliminated, so that there is only one seed per cell. Several methods have been proposed to eliminate redundant seeds, although some combine regions after the watershed procedure. Given that mitotic and glia cells do not form close clusters, we found a simple solution that was good enough to reject redundant seeds. Every seed consists of a set of voxels. If a cell has two or more seeds, this is due to shape irregularities that make an object have more than one peak. However, these peaks are not very high and when domes are found, these additional seeds are formed by a very small quantity of voxels. Therefore, we found that rejecting seeds formed by less than 20 voxels resulted in the elimination of most redundant seeds. The minimum value of a seed is not a very critical value, given that it was found that most of the spurious seeds are constituted by a maximum of 10 voxels and true seeds by a minimum of 100 voxels. Once the seeds have been selected the 3D watershed employing the Image Foresting Transform (IFT) was finally applied [26]. Watershed allowed separating very close cells (Figure 2C and Figure 3).

In order to identify the mitotic cells stained with pH3 amongst the candidate objects from the previous steps, a classification method was used (Figure 3). To determine which parameters characterize pH3 stained cells, a sample of 100 p-H3 cells obtained from one embryo were studied. Several parameters were measured and analysed statistically. pH3 stains mitotic chromosomes, resulting in objects of irregular shape. Volume was found to be the best identifier. Minimum volume was set as the parameter to reject candidate objects. A 3D labelling method using 6-connectivity was used to label the remaining objects through all the slices of the stack. The objects with smaller volume than *V*<3.172µm^3^ were rejected. The remaining objects were identified as cells and counted. This programme was found to count just as well Repo labelled glial cells in the embryo.

## Results

To validate the proposed method, cells were counted automatically and the resulting cells were verified one by one. We used DeadEasy to analyse a stack of around 100–150 images (confocal slices) that span the entire thickness of the embryonic ventral nerve cord (VNC). DeadEasy creates a second stack where the identified objects appear in locations corresponding to the cells in the raw data stack. To validate the programme, every identified object in the processed stack is compared with the corresponding cell in the raw stack, and each object/cell is compared in each slice throughout the entire stack of images. This is important to check, for instance, that a cell is not counted as two different cells in different focal planes. Validation and counting are not done in projections of images, since this would obscure and superimpose cells. Thus, both object identification and validation are done in 3D.

DeadEasy Mito-Glia was developed for pH3. pH3 is a nuclear maker, characterised by high intensity and low background. Thus, DeadEasy Mito-Glia is likely to work also for the quantification of cells labelled with other nuclear markers. To test this, we validated the method on embryos labelled with antibodies to the general glial nuclear marker Repo. DeadEasy Mito-Glia has very low error consisting of false positives (objects wrongly counted as cells) and false negatives (missed cells) consistently below 3%, and sensitivity is very high, for both markers (Table 1). The main source of false positives is background spots; occasionally one cell may be counted as two if they appear as separate in one focal plane or slice (i.e. with pH3, where shape is irregular). The sources of false negatives are low signal intensity (i.e. objects obvious to the eye are below threshold), or too short distance between adjacent cells making them appear as one in at least one slice (e.g. two cells counted as one). Badly stained samples (Figure 2D) with low signal intensity are not processed well by DeadEasy Mito-Glia and must be discarded. DeadEasy performs consistently as it always yields the same cell number count for a given sample and it treats different samples in the same objective way. This means that constant and objective criteria are used to compare multiple samples across genotypes. Thus the method performs accurately in the quantification of, at least, mitotic cells and glia.

**Table 1:** Validation of DeadEasy MitoGlia in identifying mitotic and glia cells.

Cell type	Stacks	Cells	Real counts	False P positives (%)	False negatives (%)	Sensitivity
Mitotic pH3	10	678	685	1.31	2.33	0.98
Glia Repo	7	547	561	0	2.49	0.98

DeadEasy Mito-Glia is very fast, counting cells in a few seconds to less than 1 minute (depending on computer) per animal for each confocal stack of around 150 slices. A virtually unlimited number of samples can be loaded in one go to be quantified automatically (e.g. over-night using the plug-in “Lots DeadEasy MitoGlia”).

**Figure 4:**
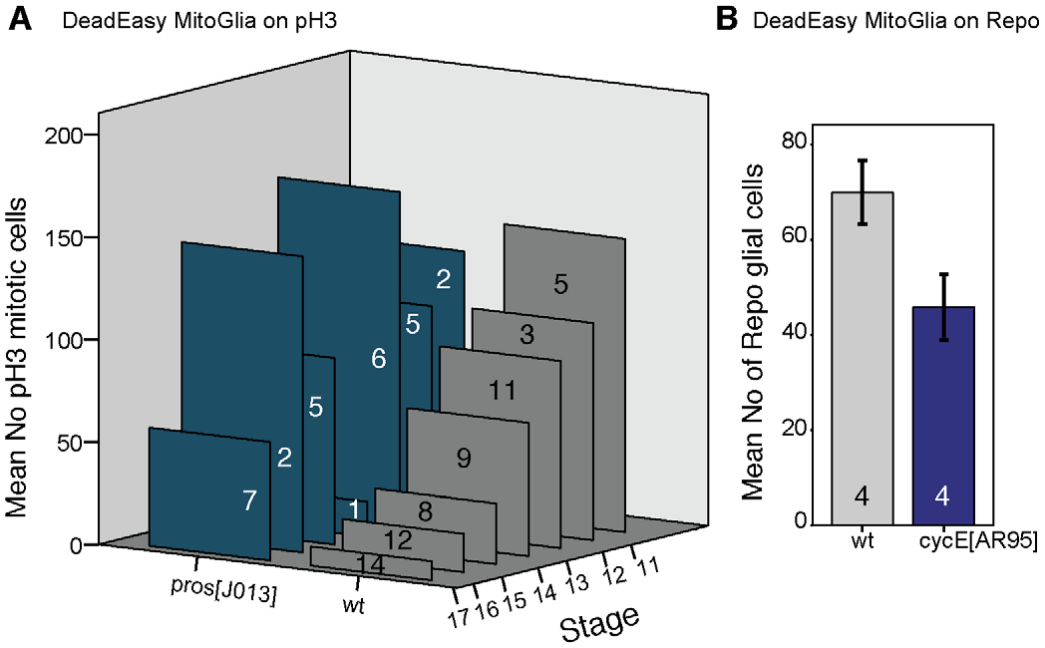
Examples of applications of DeadEasy MitoGlia to address biological questions. (A) Automatic quantification of mitotic pH3 positive cells in vivo in wild-type and *pros^J013^* null mutants, showing that proliferation increases in the mutants throughout embryogenesis. (B) Automatic quantification of Repo positive glia in wild-type and *cycE^AR95^* mutant embryos, showing a decrease in glial number when cell division is compromised. Only a subset of dorsal glia are counted here. Numbers within bars indicate sample sizes. Error bars are s.e.m.

To test the value of DeadEasy software for addressing biological questions we carried out temporal profiles of dividing cells and glia in wild-type and mutant embryos (Figure 4). We show here that DeadEasy can be used to count glia and mitotic cells in embryos. There is some variability in the number of dividing and glial cells across a population of embryos of the same age. This variability is not due to variable cell counting by DeadEasy, since it will yield exactly the same cell number regardless of how many times it is run over the same sample. Variability could be due to slight differences in the selected region of interest (also in the dorso-ventral plane); variations in immunohistochemistry outcomes; variations in confocal settings and laser life; biological variation, for instance due to changes in cell number through time/age, and regulative events. DeadEasy Mito-Glia can be used to compare cell counts between large samples of wild-type and mutant specimens, to infer gene function (Figure 4). We provide examples of the power of DeadEasy programmes to infer gene function. Prospero (Pros) is a transcription factor known for its tumour suppressor functions in neuroblasts. While *pros* has been shown to repress cell proliferation [27], it is unclear whether mutations in *pros* result in sustained cell division throughout embryogenesis. We tested this by carrying out a profile of cell proliferation throughout embryogenesis in *pros^J013^* null mutants compared to wild-type (Figure 4A). While cell division decreases markedly in wild-type embryos from stage 11 to stage 17, it continues at high levels in *pros^J013^* mutants throughout embryogenesis (Figure 4A). To test the use of DeadEasy MitoGlia to count Repo positive glia, we compared samples of embryos mutant for *cyclinE*, *cycE^AR95^* , to wild-type controls. CycE is required for the G1-S transition during cell division, thus in *cycE^AR95^* mutants the number of dorsal glial cells is lower than in wild-type (Figure 4B). These data show that DeadEasy MitoGlia is a powerful tool to count the number of embryonic dividing cells and of glial cells in vivo.

## Discussion

The method presented here is integrated into the DeadEasy programmes, a set of freely-available ImageJ plug-ins that we have developed to count automatically cells in vivo in Drosophila [1], [2]. DeadEasy Mito-Glia was developed for counting nuclei stained with a sparsely distributed die, and has been validated for the mitotic marker pH3 and the nuclear glial marker Repo in Drosophila embryos. pH3 labels the phosphorylated state of the evolutionarily conserved Histone-H3 characteristic of M-phase (mitosis) of the cell cycle. Repo labels all glial nuclei, except midline glia, in Drosophila. The nuclei stained with pH3 can be of irregular shape and nuclei stained with Repo tend to be rather regular. Both of these antibodies yield high signal intensity and low background, stain nuclei that are relatively sparsely distributed in the organism and do not tend to overlap or form large clusters in embryos. The method is very accurate for the validated markers. The above characteristics of the markers and the resulting images can be used as a guide to estimate whether DeadEasy could be used for other markers (e.g. nuclear markers such as BrdU, nuclear GFP) in Drosophila embryos. In other tissues (e.g. Drosophila epidermis, imaginal discs, larval VNC) or model organisms (e.g. fish, mouse) DeadEasy can be also be used so long as stainings of comparable qualities are used to visualise cells of comparable sizes. However, we have tested DeadEasy Mito-Glia in larvae, and it is not satisfactorily accurate at counting larval glial cells , thus we have developed another method for this purpose (Forero et al, in preparation). The parameters indicated in Table 2 and within the algorithms (e.g. threshold) used to identify the cells can be accessed through the provided interactive window (Figure 1D) and modified by the user – without any required knowledge of computation - to adapt the programme to count other cells of choice. However, in these cases the accuracy of DeadEasy may be compromised. In order to use DeadEasy MitoGlia – as well as all other DeadEasy methods – on samples for which we have not validated the programmes, the users need to go through several rounds of validation and parameter change until the desired accuracy is achieved.

**Table 2:** Parameters than can be modified and effects on performance.

DeadEasy MitoGlia (Embryos)	
**Thresholding**	This is to separate signal from noise. Increasing the standard deviations away from the mean value of the Gaussian function will decrease the number of pixels that will be considered to belong to the cells. A minimum threshold must be defined to prevent noise in darker images.
**Particle intersection**	This is to separate stained nuclei that appear to overlap in some images. Increasing the value of the percentage of intersection between two objects will increase the number of pixels required to consider that the objects do not belong to the same cell. Therefore more objects will be separated and the number of counts will increase. This programme works well for sparsely and slightly overlapping stained nuclei. For a sample type different from the default, if the stained nuclei are too close, the programme will fail at separating them.
**Minimum volume**	This is to detect the objects in 3D. Increasing the minimum volume will decrease the count, as more small particles will not be counted. For a different sample size, the minimum volume could be increased if the stained nuclei were larger than for our case.

DeadEasy counts pH3 cells, therefore it will count equally mother (just before dividing) and daughter (recently divided) cells indistinctly. This is not a problem since all samples (e.g. across multiple genotypes) are treated in exactly the same way. The only case in which this represents a problem is when daughter cells are just exiting mitosis and they are considerably smaller and are still closely adjacent to each other: in this case, DeadEasy Mito-Glia may count two cells as one. However, if the sample had been analysed a few minutes earlier, this cell would have appeared as one single (mother) cell in mitosis anyway. Thus this variability associated with the biological phenomenon of cell division is eliminated when analysing large sample sizes of the same age.

Our demonstrative analysis of *pros* mutants shows that knowledge is gained from using different methods to quantify cell number. There is no longitudinal glial hyperplasia in *pros^J013^* mutants, but instead the timing of cell division is altered, with one ectopic round of cell division at stage 12 and loss of proliferation later on [28]. Therefore the observed sustained proliferation here must be particular to neuroblasts, consistently with the notion that the functions of Pros in neuroblasts and in the longitudinal glioblast lineage differ. In *pros^J013^* mutants there is a reduction in the number of neurons that differentiate to express HB9 despite the increase in proliferation [1], confirming that in the absence of *pros* neuronal cell fate determination is aberrant in the ectopic neurons.

For its direct use as specified (mitotic or glial cells) running DeadEasy MitoGlia is extremely easy: download from our web-site, install as an ImageJ plug-in, open “ImageJ”, choose “Plug-ins” from menu, scroll down, run “DeadEasy MitoGlia”. DeadEasy MitoGlia is extremely fast, and it eliminates the tedium of manual cell counting. Because automatic counting is objective, reliable and reproducible, comparison of cell number between specimens and between genotypes is considerably more accurate with DeadEasy than with manual counting. DeadEasy programmes enable automatic cell number counts in vivo. The programmes are used as freely internet accessible ImageJ plug-ins. DeadEasy will be of interest for Drosophila researchers and for the broader scientific community.
